# The Safety and Feasibility of Enhanced Recovery after Surgery in Patients Undergoing Pancreaticoduodenectomy: An Updated Meta-Analysis

**DOI:** 10.1155/2020/7401276

**Published:** 2020-05-08

**Authors:** You-Meng Sun, Ying Wang, Yi-Xin Mao, Wei Wang

**Affiliations:** The First Affiliated Hospital, Zhejiang University School of Medicine, Hangzhou, 310003 Zhejiang Province, China

## Abstract

**Background:**

Enhanced recovery after surgery (ERAS) is a multimodal, multidisciplinary, evidence-based approach to care for surgical patients and aims at optimizing the perioperative management and outcomes. The ERAS approach was first implemented in colorectal surgery patients; however, the reported applications in pancreatoduodenectomy patients are limited. In recent years, studies on ERAS for patients undergoing pancreaticoduodenectomy have been published. The accumulation of new randomized controlled trials and high-quality case-control studies stimulated us to update the analysis. Our study comprehensively collected data to provide the best evidence summary for the clinic.

**Aim:**

To evaluate the safety and feasibility of enhanced recovery after surgery in the perioperative management of pancreatoduodenectomy patients.

**Methods:**

A systematic literature search of PubMed, Embase, and the Cochrane Library was performed up to July 2019. All randomized controlled trials and case-control studies that applied ERAS for patients undergoing pancreaticoduodenectomy were considered for inclusion in this study. The patients were divided into two groups: patients who received the ERAS perioperative management approach were defined as the ERAS group and patients who received the traditional perioperative management approach were defined as the control group. All statistical analyses were conducted using the Revman5.3 software, and the outcomes were calculated as odds ratios or weighted mean differences with their corresponding 95% confidence intervals. A funnel plot was created to assess publication bias. Subgroup and sensitivity analyses were performed to explore the sources of heterogeneity.

**Results:**

A total of 20 studies involving 3613 patients (1914 patients in the ERAS group vs. 1699 patients in the control group) were included in this study. Among the 20 studies, 4 were randomized controlled trials, and 16 were case-control studies. The overall postoperative complication rate was significantly lower in the ERAS group (OR = 0.62, 95% CI: 0.53-0.74, *P* < 0.00001) than in the control group. In addition, the minor complication rate (Clavien-Dindo I-II) was also lower in the ERAS group (OR = 0.70, 95% CI: 0.58-0.86, *P* = 0.0005). The patients in the ERAS group had a lower incidence of delayed gastric emptying (OR = 0.51, 95% CI: 0.42-0.63, *P* < 0.00001) and shorter length of hospital stay (WMD = −4.27, 95% CI: -4.81~-3.73, *P* < 0.00001) than in the control group. The rates of pancreatic fistula (regardless of Grade A/B/C), wound infections, abdominal abscesses, readmission, reoperation, and morbidity were not significantly different between the two groups.

**Conclusion:**

The ERAS approach is safe and effective in the perioperative management of patients undergoing pancreaticoduodenectomy and helps to accelerate the postoperative recovery and improve prognosis.

## 1. Introduction

Enhanced recovery after surgery (ERAS) was first proposed by Kehlet [1] from the University of Copenhagen in Denmark in 1997. ERAS is a multimodal, multidisciplinary, evidence-based approach to care for surgical patients and aims at optimizing the perioperative management and outcomes. The aim of this approach is to alleviate the patient's surgical stress response, reduce postoperative complications, promote functional recovery, shorten the length of hospital stay, and achieve rapid recovery [2]. The ERAS approach was first implemented in colorectal surgery patients [3] and has now been widely applied all over the world. Different ERAS guidelines or consensuses have been published in multiple areas such as rectal/pelvic surgery [4], pancreaticoduodenectomy [5], radical cystectomy [6], gastrointestinal surgery [7], lung surgery [8], colorectal surgery [9], gynecologic/oncology [10], cesarean delivery [11], and cardiac surgery [12]. Pancreaticoduodenectomy, also known as the Whipple procedure, is the only potentially curative intervention for malignant tumors of the pancreas and duodenal ampulla.

Pancreaticoduodenectomy is often considered one of the most challenging operations in general surgery, and it takes a long time for patients to recover. In recent years, a series of studies on ERAS, which has been widely applied in patients undergoing pancreaticoduodenectomy, have been published [13, 14]. These studies suggested that implementation of ERAS programs in patients undergoing pancreaticoduodenectomy is a safe and effective approach to promote postoperative recovery. The accumulation of new randomized controlled trials and high-quality case-control studies stimulated us to update the analysis. Our study comprehensively collects data to provide the best evidence summary for the clinic.

## 2. Materials and Methods

### 2.1. Literature Search

A systematic literature search of PubMed, Embase, and the Cochrane Library was performed to collect randomized controlled trials or case-control studies that applied the ERAS approach in the perioperative management of patients undergoing pancreaticoduodenectomy, and all of the studies were performed from the inception of the database to July 2019. The search terms and relative variants were as follows: “Pancreaticoduodenectomy,” “Pancreatectomy,” “Duodenopancreatectomy,” “ERAS,” “enhanced recovery after surgery,” “FTS,” “fast track surgery,” “accelerated recovery surgery,” “rapid recovery surgery,” “clinical pathway,” and “critical pathway.” The PubMed search strategy is detailed in Table S1.

### 2.2. Inclusion Criteria

1. Type of study: randomized controlled trials or case-control studies, with the language limited to English
2. Participants: patients aged ≥18 years who underwent elective pancreaticoduodenectomy or pylorus-preserving pancreaticoduodenectomy and patients who underwent a Whipple procedure, regardless of sex or nationality
3. Interventions: patients who received the ERAS perioperative management approach defined as the experimental group and patients who received the traditional perioperative management approach defined as the control group. According to the guidelines [5] for the perioperative care of pancreaticoduodenectomy patients issued in 2012, at least 9 of 27 recommendation items should be implemented in the ERAS group.

### 2.3. Exclusion Criteria

1. Full text of the article was not available
2. Republished
3. Focus on palliative surgery, emergency surgery, or laparoscopic pancreaticoduodenectomy
4. According to the MINORS [15] scoring standard, the study quality score was lower than 13 points
5. Unextractable useful outcomes.

### 2.4. Data Extraction and Quality Assessment

Data were extracted by two investigators independently, the following information was extracted from each eligible study: name of the author, year of publication, country, study design, the number of patients in the ERAS group and control group, the total sample size, demographic data, type of surgery, interventions, outcomes, and so on. Two investigators independently evaluated the quality of the articles, and any differences were unified through discussion and a consult with a third investigator. The quality of the randomized controlled trials was evaluated by the Cochrane risk assessment tool and the quality of the case-control studies were evaluated with the methodological index for nonrandomized controlled studies [15].

### 2.5. Outcomes of Interest

The primary outcome was the overall postoperative complications, and the secondary outcomes are the rates of pancreatic fistula, delayed gastric emptying, incision infections, abdominal abscesses, readmission, reoperation, and mortality as well as the length of hospital stay. Pancreatic fistula [16] was defined using the International Pancreatic Fistula Study Group (ISGPF) guidelines and was described as a drain output of any measurable volume of fluid on or after postoperative day 3 with an amylase content greater than 3 times the serum amylase activity. Delayed gastric emptying [17] was defined according to the International Study Of Pancreatic Surgery (ISGPS) as the need to maintain a nasogastric tube (NGT) for >3 d, postoperative vomiting for 3 days with a NGT or for 7 days while not being able to tolerate a solid diet. The length of hospital stay referred to the time from the date of surgery to the date of discharge. The overall postoperative complications included any complications within 30 days from surgery to discharge, and the severity was graded by the Clavien-Dindo system [18] into minor complications (Grades I-II) and moderate and major complications (Grades III-IV). Readmission was defined as a readmission within 30 days of discharge. Reoperation was defined as a reoperation required for patients with complications or for other reasons within 30 days after discharge. Mortality was defined as death within 30 days from surgery to discharge.

### 2.6. Statistical Analysis

Statistical analysis was performed with the Revman5.3 software, and the outcomes were calculated as odds ratios (ORs) or weighted mean differences (WMDs) with their corresponding 95% confidence intervals (CIs). The heterogeneity between studies was analyzed by the chi-squared test, with the test level being *α* = 0.05, and *I*^2^ was used to measure the heterogeneity. Subgroup analyses were performed by separately analyzing only the Western countries, Eastern countries, case-control studies, and randomized controlled trials to explore the potential sources of heterogeneity. The overall postoperative complications were taken as the outcome, and sensitivity analyses were carried out by excluding one study in each round. In addition, a funnel plot was created to assess the publication bias based on the incidence of postoperative complications, pancreatic fistula, delayed gastric emptying, and mortality.

## 3. Results

### 3.1. Study Selection

**Figure 1:**
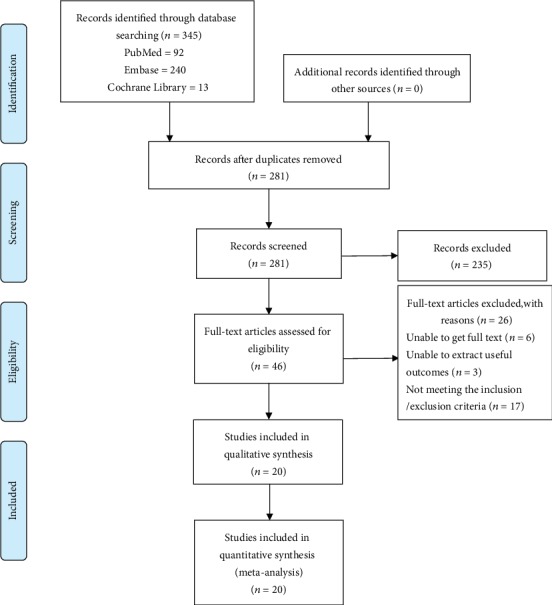
PRISMA flow diagram of the included studies eligible for meta-analysis.

According to the previous search strategy, a total of 345 records were retrieved from the online database up to July 2019. After removing the duplicates, 281 records remained, and 235 records were excluded by reviewing the title and abstract. After reading the remaining 46 records carefully, 26 records were removed for many reasons. Ultimately, 20 full-text studies [19–38] met the study inclusion criteria and were incorporated in this meta-analysis. A flow chart of the inclusion criteria to determine studies suitable for this meta-analysis is as follows (Figure 1). A total of 3613 patients (1914 patients in the ERAS group vs. 1699 patients in the control group) were involved, and among the 20 studies, 4 were randomized controlled trials [33, 35, 37, 38], and 16 were case-control studies [19–32, 34, 36]. The characteristics and quality assessments of the included studies are summarized in Table 1, and the demographics of the included studies are shown in Table S2.

### 3.2. Perioperative Interventions in the ERAS Group

An international working group, the European Association for Clinical Nutrition and Metabolism, constructed an ERAS Society recommendation in 2012 [5] and provided a comprehensive, evidence-based framework that aims at optimizing perioperative care for pancreatoduodenectomy patients. The evidence and recommendations were classified according to the GRADE system, and the quality of the evidence was divided into four levels: “high,” “moderate,” “low,” or “very low.” The recommendations were graded as “strong” or “weak.” The following 27 items were included: (1) preoperative counseling, (2) perioperative biliary drainage, (3) preoperative smoking and alcohol consumption, (4) preoperative nutrition, (5) perioperative oral immunonutrition (IN), (6) oral bowel preparation, (7) preoperative fasting and preoperative treatment with carbohydrates, (8) preanesthetic medication, (9) antithrombotic prophylaxis, (10) antimicrobial prophylaxis and skin preparation, (11) epidural analgesia, (12) intravenous analgesia, (13) wound catheters and transversus abdominis plane block, (14) postoperative nausea and vomiting (PONV), (15) incisions, (16) avoiding hypothermia, (17) postoperative glycaemic control, (18) nasogastric intubation, (19) fluid balance, (20) perianastomotic drain, (21) somatostatin analogues, (22) urinary drainage, (23) delayed gastric emptying (DGE), (24) stimulation of bowel movements, (25) postoperative artificial nutrition, (26) early and scheduled mobilization, and (27) audits. Among the included studies, 17 articles [22–38] that were published after 2012 had a high compliance rate with the interventions of different ERAS elements, and 3 articles [19–21] that were issued before 2012 met the standards of the interventions that applied more than 9 items and were also considered in our meta-analysis. The detailed elements of the ERAS approach of each study are shown in Table S3.

### 3.3. Quality Assessment of the Included Studies

Four randomized controlled trials [33, 35, 37, 38] all mentioned the generation of random sequences, one [37] of them referred to the grouping method, and the other [35] described the loss to follow-up. None of the subjects, intervention implementers, or outcome measure evaluators were blinded in these studies. The quality grades were Bs for all of the included randomized controlled trials. The MINORS scores of 16 case-control studies [19–32, 34, 36] were ≥13 points. The bias risk assessment form for the included studies is shown in Table S4.

### 3.4. Meta-Analysis Outcomes

#### 3.4.1. Primary Outcome

**Figure 2:**
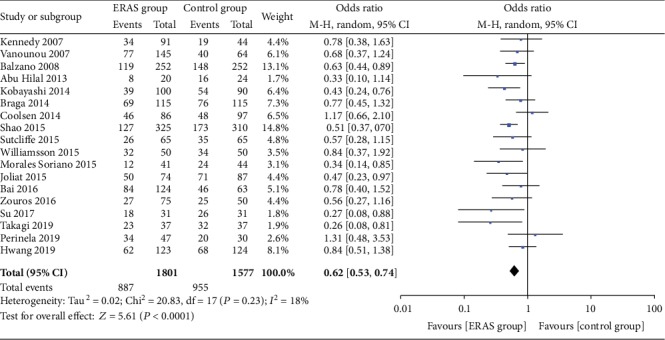
Forest plots demonstrating the outcomes of overall postoperative complications.

**Figure 3:**
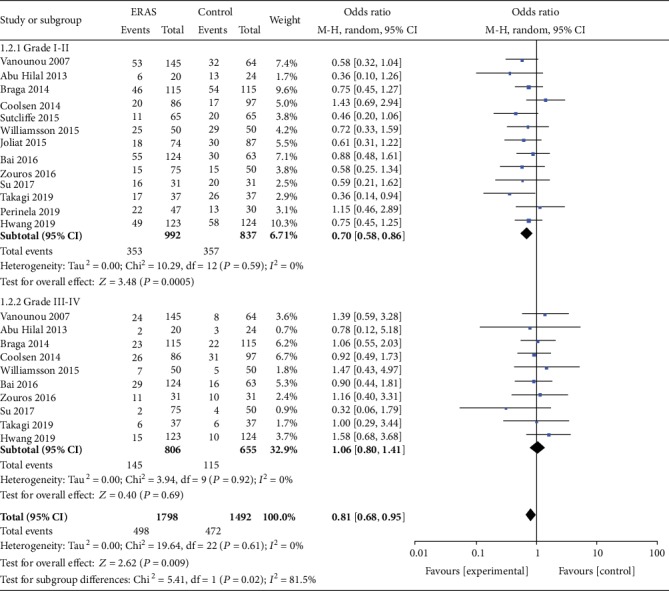
Forest plots demonstrating the outcomes of overall postoperative complications based on the classification of Clavien-Dindo.

*(1) Overall Postoperative Complications*. A total of 18 studies [19–32, 34–36, 38] reported the incidence of overall postoperative complications. The meta-analysis results showed that the rate of overall postoperative complications was significantly lower in the ERAS group (OR = 0.62, 95% CI: 0.53-0.74, *P* < 0.00001; Figure 2). In addition, 13 studies [20, 22, 24, 25, 27, 28, 30–32, 34–36, 38] classified the incidence of overall postoperative complications based on the Clavien-Dindo severity definitions. The incidence of minor complications (Clavien-Dindo I-II) was lower in the ERAS group than in the control group (OR = 0.70, 95% CI: 0.58-0.86, *P* = 0.0005); however, there were no statistically significant differences in the moderate and severe complications (Clavien-Dindo III-IV; OR = 1.06, 95%CI = 0.80 − 1.41, *P* = 0.69; Figure 3) between the two groups.

### 3.5. Secondary Outcomes

#### 3.5.1. Pancreatic Fistula

**Figure 4:**
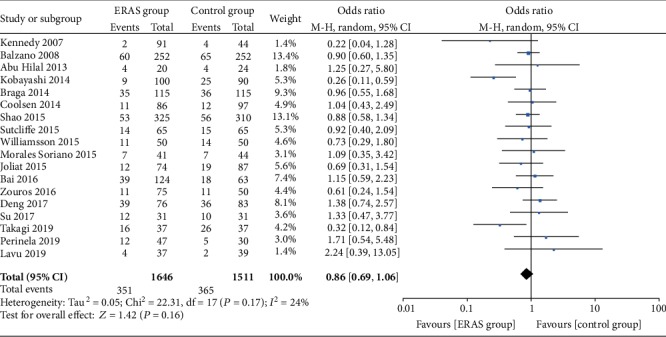
Forest plots demonstrating the outcomes of pancreatic fistulas.

**Figure 5:**
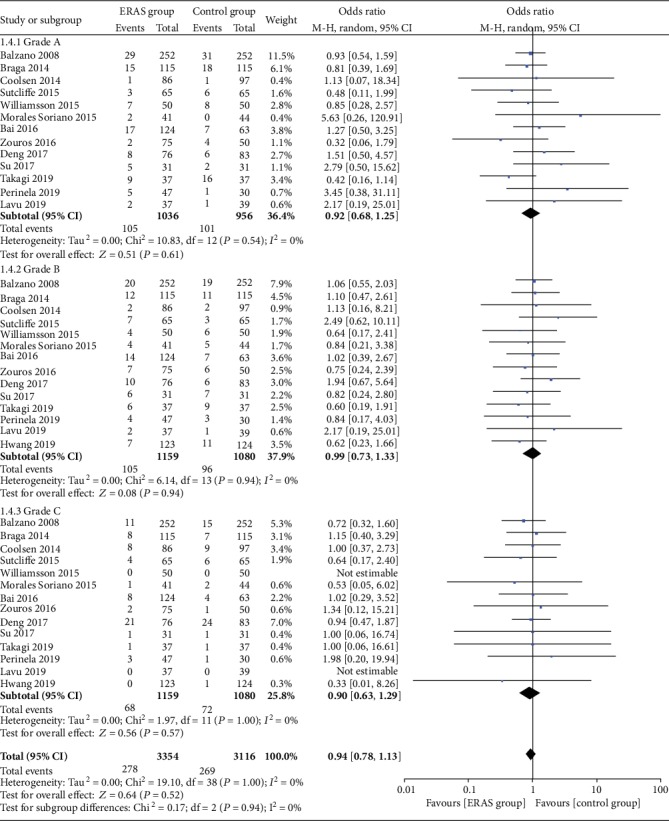
Forest plots demonstrating the outcomes of pancreatic fistula according to the definition of International Pancreatic Fistula Study Group.

A total of 18 studies [19, 21–37] also reported the incidence of pancreatic fistula. The meta-analysis showed no significant difference between the two groups (OR = 0.86, 95% CI: 0.69-1.06, *P* = 0.16; Figure 4). Furthermore, 13 studies [21, 24, 25, 27–29, 31–37] subdivided the severity of pancreatic fistulas according to the pancreatic fistula grading standard (A/B/C) developed by the International Pancreatic Fistula Study Group (ISGPF). The combined analysis also demonstrated that there were no significant differences in Grade A (OR = 0.92, 95% CI: 0.68-1.25, *P* = 0.61), Grade B (OR = 0.99, 95% CI: 0.73-1.33, *P* = 0.94), and Grade C (OR = 0.90, 95% CI: 0.63-1.29, *P* = 0.57; Figure 5) pancreatic fistulas between the two groups.

#### 3.5.2. Delayed Gastric Emptying

**Figure 6:**
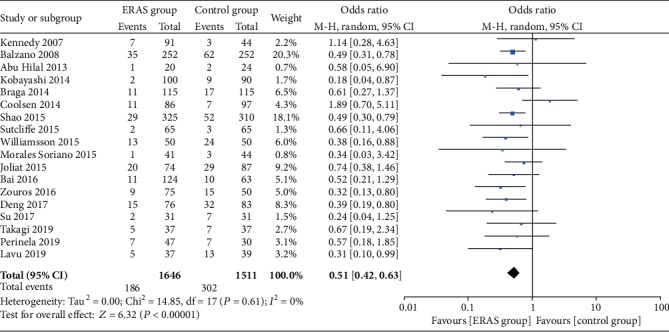
Forest plots demonstrating the outcomes of delayed gastric emptying.

A total of 18 studies [19, 21–37] reported the incidence of delayed gastric emptying, and a total of 3157 patients were involved. Compared to the control group, the ERAS group had a significantly lower incidence of delayed gastric emptying (OR = 0.51, 95% CI: 0.42-0.63, *P* < 0.00001; Figure 6).

#### 3.5.3. Length of Hospital Stay

**Figure 7:**
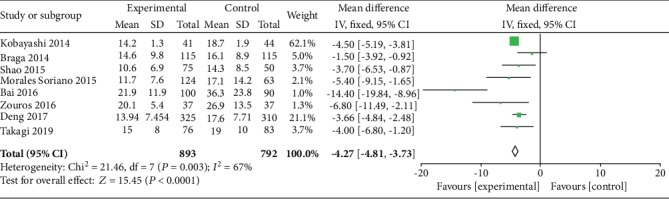
Forest plots demonstrating the outcomes of length of hospital stay.

Eight studies [23, 24, 26, 29, 31–33, 35] reported the length of hospital stay, and 1685 patients were involved. The current results revealed that the ERAS group had a significantly shorter length of hospital stay than the control group (WMD = −4.27, 95% CI: -4.81~-3.73, *P* < 0.00001; Figure 7).

#### 3.5.4. Other Outcomes

**Figure 8:**
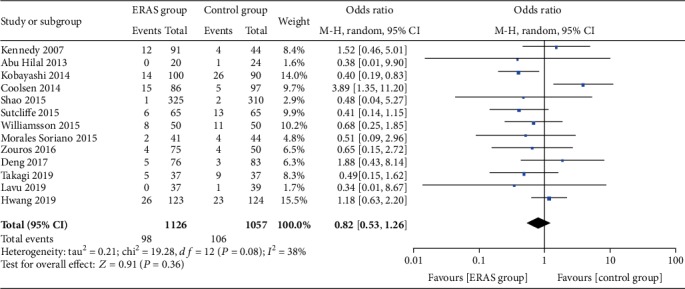
Forest plots demonstrating the outcomes of wound infection.

**Figure 9:**
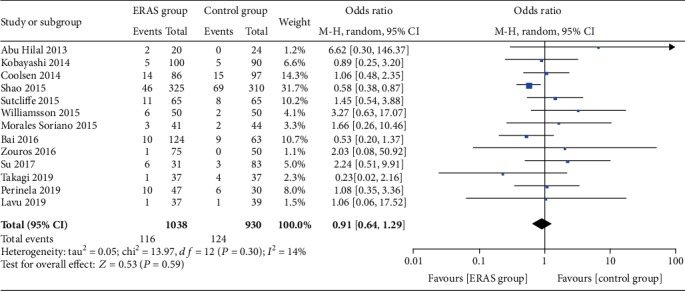
Forest plots demonstrating the outcomes of abdominal abscesses.

**Figure 10:**
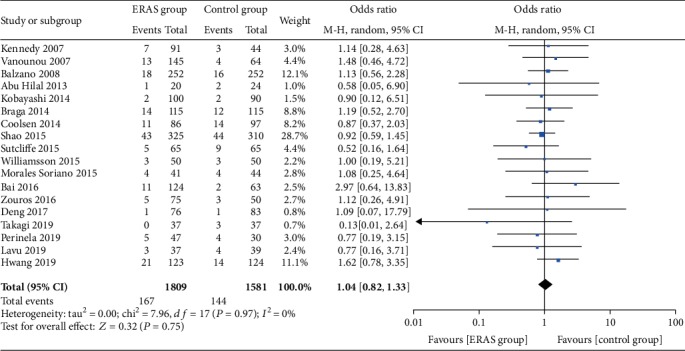
Forest plots demonstrating the outcomes of readmission.

**Figure 11:**
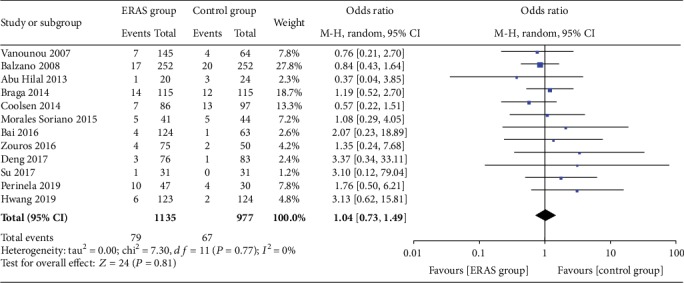
Forest plots demonstrating the outcomes of reoperation.

**Figure 12:**
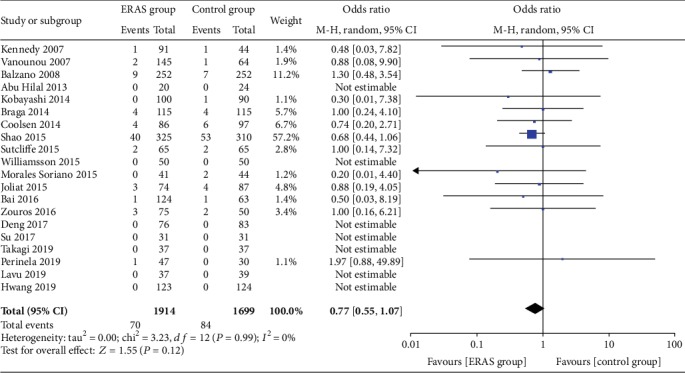
Forest plots demonstrating the outcomes of morbidity.

The rate of wound infections (OR = 0.82, 95% CI: 0.53-1.26, *P* = 0.36; Figure 8), the rate of abdominal abscesses (OR = 0.91, 95% CI: 0.64-1.29, *P* = 0.59; Figure 9), readmission rates (OR = 1.04, 95% CI: 0.82-1.33, *P* = 0.75; Figure 10), reoperation rates (OR = 1.04, 95% CI: 0.73-1.49, *P* = 0.81; Figure 11), and morbidity rates (OR = 0.77, 95% CI: 0.55-1.07, *P* = 0.12; Figure 12) were not significantly different between the two groups.

#### 3.5.5. Subgroup Analysis and Sensitivity Analysis

Subgroup analysis was performed by separately analyzing only Western countries [19–22, 24, 25, 27–30, 32, 36, 37], Eastern countries [23, 26, 31, 33–35, 38], randomized controlled trials [33, 35, 37, 38], and case-control studies [19–32, 34, 36]. All of the subgroups produced outcomes consistent with the overall outcomes, except the RCT subgroup. We considered that the main reason contributing to the difference was an insufficient number of RCTs. When the analysis focused only on Western countries, the heterogeneity between studies dropped dramatically or even disappeared. However, among Eastern countries, the heterogeneity was obviously increased compared to the overall results. When only the case-control studies were analyzed, both the outcomes and the heterogeneity of each subgroup were very close to the overall results. All of the results of the subgroup analyses are displayed in Table 2.

The overall postoperative complications were taken as the outcome, and sensitivity analyses were carried out by excluding one study in each round. Sensitivity analysis showed that no knockout of every study had a particularly large effect on the results. It is worth mentioning that when the study of Coolsen et al. [25] was removed, the heterogeneity was eliminated. All of the results of the sensitivity analyses are presented in Table S5.

#### 3.5.6. Publication Bias

**Figure 13:**
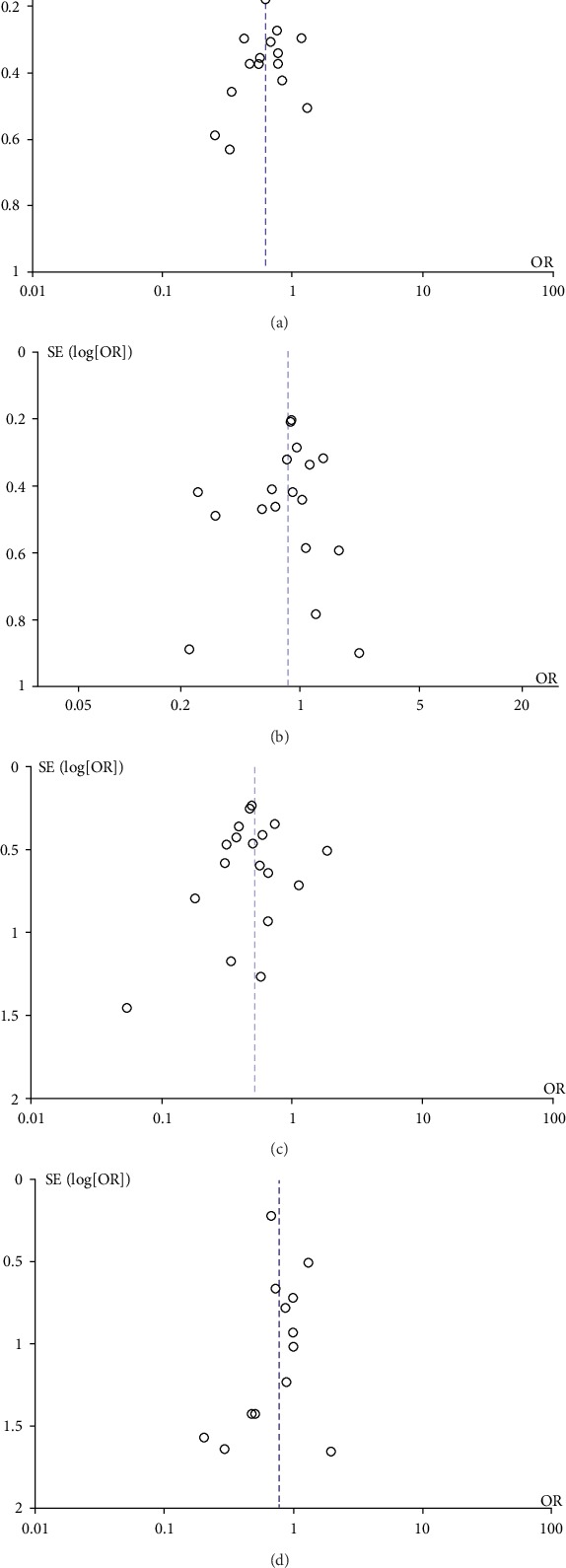
Funnel plot of overall postoperative complications (a), pancreatic fistula (b), delayed gastric emptying (c), and morbidity (d) in all included studies. SE: standard deviation; OR: odds ratio.

The incidence of overall postoperative complications, pancreatic fistulas, and delayed gastric emptying and mortality rates were drawn as funnel plots to evaluate the potential publication bias. The funnel plots indicated a left-right symmetrical distribution, and publication bias had little impact on the meta-analysis (Figure 13).

## 4. Discussion

Pancreaticoduodenectomy is the main or perhaps the only potentially curative treatment for malignant tumors of the pancreas and duodenal ampulla. However, pancreaticoduodenectomy is complicated and has a high rate of postoperative complications. With the development of medical technology, the introduction of various advanced instruments and equipment, the continuous optimization of perioperative management, and the tendency for centralization in pancreaticoduodenectomy, the mortality rate is approximately 5% [39, 40], and even in some high-volume centers, the mortality rate has even been reduced to 1-2% [41, 42]. However, the rate of overall postoperative complications is still as high as 30%-60% [43–45]. In particular, complications such as pancreatic fistula, delayed gastric emptying, wound infections, and abdominal abscesses prolong the length of hospital stay and increase the risk for readmission, reoperation, and even death. Therefore, higher requirements need to be put forward for more refined perioperative management in the clinic. The ERAS is safe and has been effectively implemented in colorectal surgery; currently, the approach has been widely accepted and internationally applied to patients undergoing pancreaticoduodenectomy.

ERAS is a multimodal, multidisciplinary, evidence-based approach to care for surgical patients and aims at optimizing the perioperative management and outcomes. The core of the ERAS concept is to reduce the patient's fasting time, provide preoperative treatment with carbohydrates, provide multimodal analgesia, administer goal-directed fluid therapy, promote early feeding, promote early extubation, and implement early mobilization to alleviate the patient's surgical stress responses, reduce postoperative complications, promote function recovery, shorten the length of hospital stay, and achieve rapid recovery. ERAS requires multidisciplinary cooperation among the departments of surgery, anesthesia, nursing, nutrition, pain, and rehabilitation.

Our meta-analysis included the latest 20 studies [19–38] from 2007 to 2019 to evaluate the safety and efficacy of ERAS in patients undergoing pancreaticoduodenectomy. Compared with the previous meta-analysis [13, 14], our study covered more randomized controlled trials, thus conferring a higher grade of medical evidence to support the outcomes. In our study, the baseline characteristics of the ERAS group and control group were consistent, and strict inclusion and exclusion criteria were followed. All of the studies elaborated on standardized definitions for the outcomes; there was no or little heterogeneity in the observation of most outcomes, and the subgroup analysis and sensitivity analysis also had no effect on the outcomes. Our study shows that ERAS can reduce the overall postoperative complication rates, particularly with respect to the minor complication rate, reduce the incidence of DGE, and shorten the length of hospital stay. The incidence of moderate and serious complications, incidence of pancreatic fistula (regardless of Grade A/B/C), incidence of incision infections, incidence of abdominal abscesses, readmission rate, reoperation rates, and mortality rates were not significantly different between the two groups (*P* > 0.05), which were confirmatory of previous analyses [13, 14].

Five of the articles [24, 31, 32, 34, 35] we included mentioned patient compliance. Braga et al. [24] found that the compliance with preoperative and intraoperative ERAS items was higher (84%-100%), while compliance with postoperative ERAS items was relatively low (38%-66%). The subgroup analysis showed that better compliance was observed in patients without complications than in those with complications. Furthermore, patient compliance gradually decreased as more severe postoperative complications occurred. Bai et al. [31] demonstrated that the rate of preoperative compliance with ERAS core elements was 74.8%-100%; however, the rate of postoperative compliance was 60.4%-95.2%. Similarly, Zouros et al. [32] reported that the rate of compliance with various elements ranged from 74.7% to 100% and that patients with no complications or minor postoperative complications had a higher adherence rate to ERAS and shorter hospital stay than those with major complications. Su et al. [34] showed that the compliance with preoperative and intraoperative ERAS protocol elements was 71%-100%, but the compliance with postoperative was decreased to 58%-84%. Takagi et al. [35] found that 84% of the patients followed the preoperative and intraoperative ERAS protocol, while only 30% of the patients followed the postoperative ERAS pathway. Wong et al. [46] investigated the implementation of protocols based on the ERAS concept in the perioperative period of liver surgery in 11 HPB centers in Europe, and the results showed that the compliance rate for the postoperative ERAS protocol was not promising. A survey [47] of 2352 colorectal surgery patients who were treated with the ERAS protocol in 13 centers from 6 countries showed that a higher compliance rate was associated with a lower postoperative complication rate and shorter length of hospital stay. Therefore, improvements in patient compliance are essential to guaranteeing that ERAS to be implemented in practice.

Several potential limitations of the present analysis should be acknowledged. First, the majority of the studies included were retrospective case-control studies, which may lead to selection bias and recall bias. None of the randomized controlled trials featured blinding for the subjects, intervention operators, or outcome measurers, which may lead to implementation bias and measurement bias. Second, the specific ERAS protocols vary among different studies, and they included a minimum of 9 items and a maximum of 25 items; additionally, patient compliance was hard to control, which may lead to clinical heterogeneity. It is worth mentioning that the implementation of a blinding method for the ERAS protocol itself is not feasible; this is also the main reason why the previous meta-analysis only included case-control studies and the main factor that led to the moderate quality of RCTs in our study.

In conclusion, the ERAS approach is safe and effective in the perioperative management of pancreatoduodenectomy patients, and it can accelerate the postoperative recovery, promote better recovery with respect to gastrointestinal function, and shorten the length of hospital stay. Large-sample, multicenter, prospective research is needed to provide more solid evidence. Currently, the ERAS guidelines in many fields advocate for the selection of minimally invasive surgery, and since laparoscopic pancreaticoduodenectomy is maturing in hepatobiliary surgery, we look forward to more literature that reports the application of ERAS for LPD patients. In clinical practice, an ERAS team is required to ensure that each item is implemented, provide precise and individualized patient management, improve patient compliance, and promote patient recovery.

## Figures and Tables

**Table 1:** Characteristics and quality assessment of the included studies.

Study	Year	Country	Study design	Sample size		Total	MINORS score
				ERAS group	Control group		
Kennedy et al.	2007	United States	Case-control study	91	44	135	15/24
Vanounou et al.	2007	United States	Case-control study	145	64	209	13/24
Balzano et al.	2008	Italy	Case-control study	252	252	504	15/24
Abu Hilal et al.	2013	Britain	Case-control study	20	24	44	14/24
Kobayashi et al.	2014	Japan	Case-control study	100	90	190	13/24
Braga et al.	2014	Italy	Case-control study	115	115	230	18/24
Coolsen et al.	2014	Netherlands	Case-control study	86	97	183	15/24
Shao et al.	2015	China	Case-control study	325	310	635	14/24
Sutcliffe et al.	2015	Britain	Case-control study	65	65	130	14/24
Williamsson et al.	2015	Sweden	Case-control study	50	50	100	16/24
Morales Soriano et al.	2015	Spain	Case-control study	41	44	85	16/24
Joliat et al.	2015	Switzerland	Case-control study	74	87	161	15/24
Bai et al.	2016	China	Case-control study	124	63	187	15/24
Zouros et al.	2016	Greece	Case-control study	75	50	125	16/24
Deng et al.	2017	China	RCT	76	83	159	^∗^
Su et al.	2017	China	Case-control study	31	31	62	15/24
Takagi et al.	2019	Japan	RCT	37	37	74	^∗^
Perinela et al.	2019	France	Case-control study	47	30	77	19/24
Lavu et al.	2019	United States	RCT	37	39	76	^∗^
Hwang et al.	2019	Korea	RCT	123	124	247	^∗^

RCT: randomized controlled trial; MINORS: methodological index for nonrandomized studies. ^∗^Unconformity to MINORS score criteria.

**Table 2:** Results of subgroup analysis.

Outcome of interest	No. of studies	No. of patients	OR/WMD	95% CI	*P* value	Heterogeneity *P* value	*I* ^2^%
Studies in Western countries							
PF	12	1850	0.89	0.71-1.13	0.35	0.85	0
DGE	12	1850	0.56	0.43-0.73	<0.0001	0.43	1
Overall morbidity	12	1983	0.68	0.56-0.82	<0.0001	0.46	0
LOS	3	491	-3.30	-5.17, -1.44	0.0005	0.06	63
Readmission	12	1898	0.99	0.72-1.38	0.97	1.00	0
Reoperation	8	1457	0.92	0.63-1.34	0.67	0.86	0
Mortality	13	2059	0.95	0.56-1.61	0.84	0.99	0
Studies in Eastern countries							
PF	6	1307	0.76	0.46-1.27	0.30	0.008	68
DGE	6	1307	0.44	0.32-0.62	<0.00001	0.77	0
Overall morbidity	6	1395	0.54	0.39-0.74	<0.00001	0.17	36
LOS	5	1194	-4.36	-4.93, -3.79	<0.00001	0.005	73
Readmission	6	1492	1.12	0.75-1.66	0.58	0.39	4
Reoperation	4	655	2.88	0.99-8.41	0.05	0.99	0
Mortality	7	1554	0.67	0.43-1.03	0.07	0.86	0
Case-control studies						
PF	15	2848	0.85	0.70-1.04	0.11	0.38	7
DGE	15	2848	0.53	0.43-0.61	<0.0001	0.50	0
Overall morbidity	16	3057	0.62	0.52-0.73	<0.00001	0.31	13
LOS	6	891	-4.46	-5.08, -3.83	<0.00001	0.001	75
Readmission	14	2834	1.01	0.77-1.31	0.96	0.98	0
Reoperation	10	1706	0.96	0.66-1.38	0.82	0.89	0
Mortality	16	3057	0.77	0.55-1.07	0.12	0.99	0
RCT							
PF	3	309	0.91	0.30-2.79	0.87	0.03	72
DGE	3	309	0.41	0.24-0.71	0.002	0.66	0
Overall morbidity	2	321	0.52	0.17-1.62	0.26	0.07	71
LOS	2	794	-3.71	-4.80, -2.62	<0.00001	0.83	0
Readmission	4	556	1.24	0.64-2.39	0.52	0.38	2
Reoperation	2	406	3.21	0.86-12.03	0.08	0.96	0

OR: odds ratio; WMD: weighted mean difference; CI: confidence interval; PF: pancreatic fistula; DGE: delayed gastric emptying; LOS: length of hospital stay.

## Data Availability

The datasets used and/or analyzed during the current study are available from the corresponding author on reasonable request.

## Additional Points

### Core Tip

This is a meta-analysis with the latest 20 studies up to July 2019 that evaluates the safety and efficacy of ERAS in patients undergoing pancreaticoduodenectomy. Compared to the previous meta-analysis that only included case-control studies, our study first covered randomized controlled trials and confers a higher grade of medical evidence to support the outcomes. We applied strict inclusion and exclusion criteria and demanded higher standards on the quality of the included studies. In addition, our study elaborated on the standardized definitions of outcomes and comprehensively summarized the perioperative interventions in the ERAS group to ensure that the conclusion is highly reliable.

### PRISMA 2009 Checklist Statement

The authors have read the PRISMA 2009 Checklist, and the manuscript was prepared and revised according to the PRISMA 2009 Checklist.

## Supplementary Materials

Supplementary Materials Table S1: PubMed Search Strategy. Table S2: demographics of the included studies. Table S3: elements of enhanced recovery after surgery. Table S4: bias risk assessment form of the included studies. Table S5: results of sensitivity analysis.
