# Supplementary material for: The Safety and Feasibility of Enhanced Recovery after Surgery in Patients Undergoing Pancreaticoduodenectomy: An Updated Meta-Analysis
Source: Biomed Res Int. 2020 May 8;2020:7401276. doi: 10.1155/2020/7401276 (PMC7232716; doi:10.1155/2020/7401276)
Supplement: Supplementary Materials — Table S1: PubMed Search Strategy. Table S2: demographics of the included studies. Table S3: elements of enhanced recovery after surgery. Table S4: bias risk assessment form of the included studies. Table S5: results of sensitivity analysis. [file 7401276.f1.docx]

| **Table S1 PubMed Search Strategy** | | |
| --- | --- | --- |
| **Step**  **Query Items found** | | |
| #1: | Search "Pancreaticoduodenectomy"[Mesh] | 7585 |
| #2: | Search((((Pancreaticoduodenectomies[Title/Abstract]) OR Pancreatoduodenectomy[Title/Abstract]) OR Pancreatoduodenectomies[Title/Abstract]) OR Duodenopancreatectomy[Title/Abstract]) OR Duodenopancreatectomies[Title/Abstract] | 4009 |
| #3: | #1 OR #2 | 9407 |
| #4: | Search "Pancreatectomy"[Mesh] | 12861 |
| #5: | Search Pancreatectomies[Title/Abstract] | 627 |
| #6: | #4 OR #5 | 13055 |
| #7: | #3 OR #6 | 20633 |
| #8: | Search (((((((ERAS[Title/Abstract]) OR enhanced recovery after surgery[Title/Abstract]) OR FTS[Title/Abstract]) OR fast track surgery[Title/Abstract]) OR accelerated recovery surgery[Title/Abstract]) OR rapid recovery surgery[Title/Abstract]) OR clinical pathway[Title/Abstract]) OR critical pathway[Title/Abstract] | 9048 |
| #9： | #7 AND #8 | 92 |

| **Table S2 Demographics of the Included Studies** | | | | | | | | | | | | | |
| --- | --- | --- | --- | --- | --- | --- | --- | --- | --- | --- | --- | --- | --- |
| **Study** | **Group** | **Year** | **Female（%）** | **ASA sore（n）** | | | | | | **BMI** | **Type of surgery** | **Pancreatic duct diameter (>3mm %)** | **Pancreas texture (soft %)** |
| Kennedy2007 | ERAS | 63.9±1.3 | 55.0 | NA | | | | | | NA | PD | NA | NA |
|  | control | 61.3±2.0 | 47.0 |  |  |  |  |  |  |  |  |  |  |
| Vanounou2007 | ERAS | 64 | NA | 2 | | 53 | 84 | 0 | | NA | Whipple/ | NA | NA |
|  | control | 64 | NA | 1 | | 33 | 30 | 0 | |  | PPPD |  |  |
| Balzano2008 | ERAS | 64.3（33-88） | 58.5 | NA | | | | | | NA | PD/PPPD | 49.6 | 39.7 |
|  | control | 62.9（26-84） | 61.5 |  |  |  |  |  |  |  |  | 47.2 | 42.1 |
| Abu Hilal2013 | ERAS | 68.5（65-72） | 50.0 | 4 | | 15 | 1 | | 0 | NA | Whipple | NA | NA |
|  | control | 70（61-76） | 58.3 | 6 | | 15 | 3 | | 0 |  |  |  |  |
| Kobayashi2014 | ERAS | 67.5±10.7 | 31.1 | NA | | | | | | 21.6±3.54 | PD/PPPD/ | NA | NA |
|  | control | 65.4±10.8 | 39.0 |  |  |  |  |  |  | 25.0±4.54 | SSPD |  |  |
| Braga2014 | ERAS | 69（61-74） | 42.6 | 4 | | 88 | 23 | 0 | | 23.7（21-25） | PD/PPPD | 60.9 | 40.9 |
|  | control | 69（61-74） | 42.6 | 4 | | 82 | 29 | 0 | | 23.1（21-25） |  | 64.8 | 43.5 |
| Coolsen2014 | ERAS | 67±11† | 48.8 | NA | | | | | | NA | Whipple/ | NA | NA |
|  | control | 62±13 | 40.2 |  |  |  |  |  |  |  | PPPD |  |  |
| Shao2015 | ERAS | 56.96±11.50 | 40.3 | NA | | | | | | NA | PD/PPPD | NA | NA |
|  | control | 57.05±12.30 | 40.6 |  |  |  |  |  |  |  |  |  |  |
| Sutcliffe2015 | ERAS | 67（18-83） | 38 | NA | | | | | | 27.3±5.8 | PD/PPPD | NA | NA |
|  | control | 66（35-83） | 43 |  |  |  |  |  |  | 25.4±4.4 |  |  |  |
| Williamsson2015 | ERAS | 69（15-80） | 38 | 2 | 28 | | 20 | | 2 | 24.3（19.4-36.2） | PD | NA | NA |
|  | control | 67（25-81） | 48 | 6 | 27 | | 17 | | 0 | 25.2（16.3-33） |  |  |  |
| Morales Soriano2015 | ERAS | 61.3（44-80） | 41 | 20 | 21 | |  | | 0 | NA | PD | NA | NA |
|  | control | 66.7 | 39 | 2 | 21 | |  | | 0 |  |  |  |  |

| **Table S2 Demographics of the Included Studies** | | | | | | | | | | | | | |
| --- | --- | --- | --- | --- | --- | --- | --- | --- | --- | --- | --- | --- | --- |
| **Study** | **Group** | **Age** | **Female（%）** | **ASA sore（n）** | | | | | | **BMI** | **Type of surgery** | **Pancreatic duct diameter (>3mm %)** | **Pancreas texture (soft %)** |
| Joliat2015 | ERAS | 67.5（57-74） | 52.7 | 50 |  | | 24 | | 0 | 23.9（22.1-26.7） | PD/PPPD | NA | NA |
|  | control | 67（55-75） | 35.6 | 67 |  | | 20 | | 0 | 24.2（22.1-27.3） |  |  |  |
| Bai2016 | ERAS | 58（13） | 44.3 | 2 | | 98 | 24 | 0 | | 22.26（3.08） | PD | NA | NA |
|  | control | 57（12） | 41.3 | 1 | | 51 | 11 | 0 | | 22.44（3.46） |  |  |  |
| Zouros2016 | ERAS | 65.9±10.5 | 38.7 | 26 | 33 | | 16 | | 0 | NA | Whipple/ | 44.0 | 46.7 |
|  | control | 63.9±11.6 | 32 | 18 | 27 | | 5 | | 0 |  | PPPD | 48.0 | 48.0 |
| Deng2017 | ERAS | 54.5±12.7 | 39.5 | 54 | |  | 22 | |  | NA | PD | NA | NA |
|  | control | 51.3±15.0 | 44.6 | 64 | |  | 19 | |  |  |  |  |  |
| Su2017 | ERAS | 62±9 | 38.7 |  |  | | 5 | |  | 22.4±3.0 | PD | NA | NA |
|  | control | 61±11 | 41.9 |  |  | | 5 | |  | 22,7±2.8 |  |  |  |
| Takagi2019 | ERAS | 67.8（9.7） | 46 | 3 | | 23 | 11 | 0 | | 22.1（3.0） | PD | 48.6 | 40.5 |
|  | control | 66.8（9.3） | 46 | 6 | | 26 | 5 | 0 | | 21.7（2.8） |  | 43.2 | 29.7 |
| Perinela2019 | ERAS | 60.4±13.5 | 44 | 10 | 68 | | 19 | | 0 | 25.2±4.8 | PD | NA | NA |
|  | control | 62.3±13.5 | 51 | 19 | 41 | | 15 | | 0 | 25.2±4.2 | PPPD |  |  |
| Lavu 2019 | ERAS | 65.8±9.6 | 45.9 |  |  | | 23 | |  | 26.8±4.3 | PD | NA | NA |
|  | control | 65.0±9.3 | 53.8 |  |  | | 20 | |  | 26.1±4.9 |  |  |  |
| Hwang2019 | ERAS | 63.3±9.2 | 41.5 | 9 | 99 | | 15 | | 0 | 24.3±3.1 | PD/PPPD/ | NA | 67.5 |
|  | control | 62.9±9.2 | 34.7 | 18 | 100 | | 6 | | 0 | 24.2±3.0 | SSPD |  | 63.7 |
| ASA=American Society of Anesthesiologists, BMI=body mass index, ERAS=Enhanced recovery after surgery, NA=not available, PD=Pancreaticoduodenectomy, PPPD=Pylorus-preserving Pancreaticoduodenectomy, SSPD=Stomach-preserving Pancreaticoduodenectomy | | | | | | | | | | | | | |

| **Table S3 Elements of Enhanced recovery after surgery** | | | | | | | | | | | | | | | | | | | | | | | | | | | |
| --- | --- | --- | --- | --- | --- | --- | --- | --- | --- | --- | --- | --- | --- | --- | --- | --- | --- | --- | --- | --- | --- | --- | --- | --- | --- | --- | --- |
| **Study** | **①** | **②** | **③** | **④** | **⑤** | **⑥** | **⑦** | **⑧** | **⑨** | **⑩** | **⑪** | **⑫** | **⑬** | **⑭** | **⑮** | **⑯** | **⑰** | **⑱** | **⑲** | **⑳** | **㉑** | **㉒** | **㉓** | **㉔** | **㉕** | **㉖** | **㉗** |
| Kennedy2007 | √ |  |  |  |  |  |  |  | √ | √ |  | √ |  | √ |  |  |  | √ | √ |  |  | √ |  |  | √ | √ |  |
| Vanounou2007 | √ |  |  |  |  |  |  |  | √ | √ |  |  |  |  |  |  |  | √ | √ | √ |  | √ |  |  | √ | √ |  |
| Balzano2008 | √ |  |  |  |  |  |  |  | √ | √ | √ | √ |  | √ | √ |  |  | √ | √ | √ |  |  |  |  | √ | √ |  |
| Abu Hilal2013 | √ |  |  | √ |  |  | √ |  | √ | √ |  | √ |  | √ | √ | √ |  | √ | √ | √ |  | √ |  |  | √ | √ |  |
| Kobayashi2014 | √ | √ |  | √ |  | √ | √ |  |  |  |  |  |  |  | √ |  |  | √ |  | √ |  |  |  |  | √ |  |  |
| Braga2014 | √ | √ |  |  | √ | √ | √ | √ | √ | √ | √ |  |  | √ | √ | √ |  | √ | √ | √ |  |  |  |  | √ | √ |  |
| Coolsen2014 | √ |  |  |  |  |  | √ | √ | √ | √ | √ |  |  | √ |  | √ |  | √ | √ | √ |  | √ |  | √ | √ | √ |  |
| Shao2015 | √ |  |  |  |  |  |  |  | √ | √ | √ |  |  |  |  | √ |  | √ |  | √ |  |  | √ |  | √ | √ |  |
| Sutcliffe2015 | √ |  |  |  |  | √ | √ |  | √ | √ | √ |  |  | √ |  | √ |  | √ |  |  |  | √ |  |  | √ | √ |  |
| Williamsson2015 | √ | √ |  | √ |  |  | √ |  | √ | √ | √ | √ |  | √ | √ |  |  | √ | √ | √ |  | √ |  | √ | √ | √ |  |
| MoralesSorianon2015 | √ | √ |  |  |  | √ | √ |  | √ | √ | √ |  |  | √ |  | √ |  | √ | √ | √ |  | √ | √ | √ | √ | √ |  |
| Joliat2015 | √ |  |  |  |  | √ | √ | √ | √ | √ | √ |  |  | √ |  | √ | √ | √ | √ | √ | √ | √ |  | √ | √ | √ | √ |
| Bai2016 | √ |  |  |  |  | √ | √ |  | √ | √ |  | √ |  | √ |  |  |  | √ |  |  |  | √ |  |  | √ | √ |  |
| Zouros2016 | √ | √ |  |  |  | √ | √ | √ | √ | √ | √ | √ |  | √ |  | √ |  | √ | √ | √ | √ | √ |  | √ | √ | √ |  |
| Deng2017 | √ |  |  |  |  |  | √ | √ | √ | √ |  | √ |  | √ |  | √ |  | √ | √ | √ |  | √ |  |  | √ | √ |  |
| Su2017 | √ |  |  |  |  | √ | √ |  | √ | √ | √ | √ |  | √ |  |  |  | √ | √ |  |  | √ |  |  | √ | √ |  |
| Takagi2019 | √ | √ |  |  | √ | √ | √ | √ | √ |  | √ | √ |  |  |  | √ | √ | √ | √ | √ |  | √ |  | √ | √ | √ |  |
| Perinela2019 | √ |  |  |  | √ |  | √ | √ | √ | √ | √ | √ |  | √ |  | √ | √ | √ | √ | √ |  |  |  |  | √ | √ |  |
| Lavu2019 | √ |  |  |  |  |  |  |  | √ | √ |  | √ |  | √ |  |  |  | √ | √ | √ |  |  |  |  | √ | √ |  |
| Hwang2019 | √ | √ | √ |  | √ | √ | √ | √ | √ | √ | √ | √ | √ | √ | √ | √ | √ | √ | √ | √ |  | √ | √ | √ | √ | √ | √ |

| **Table S4 Bias Risk Assessment Form of the Included Studies** | | | | | | | | | | | | | |
| --- | --- | --- | --- | --- | --- | --- | --- | --- | --- | --- | --- | --- | --- |
| **Study** | **Items** | | | | | | | | | | | | **MINORS**  **Score/Total** |
|  | **①** | **②** | **③** | **④** | **⑤** | **⑥** | **⑦** | **⑧** | **⑨** | **⑩** | **⑪** | **⑫** |  |
| Kennedy 2007 | 2 | 2 | 2 | 1 | 0 | 2 | 0 | 0 | 2 | 0 | 2 | 2 | 15/24 |
| Vanounou 2007 | 2 | 2 | 2 | 1 | 0 | 1 | 0 | 0 | 2 | 0 | 1 | 2 | 13/24 |
| Balzano 2008 | 2 | 2 | 2 | 2 | 0 | 1 | 0 | 0 | 2 | 0 | 2 | 2 | 15/24 |
| Abu Hilal 2013 | 2 | 2 | 2 | 2 | 0 | 1 | 0 | 0 | 2 | 0 | 1 | 2 | 14/24 |
| Kobayashi 2014 | 2 | 2 | 0 | 2 | 0 | 1 | 0 | 0 | 2 | 0 | 2 | 2 | 13/24 |
| Braga 2014 | 2 | 2 | 2 | 2 | 0 | 2 | 0 | 2 | 2 | 0 | 2 | 2 | 18/24 |
| Coolsen 2014 | 2 | 2 | 2 | 2 | 0 | 1 | 0 | 0 | 2 | 0 | 2 | 2 | 15/24 |
| Shao 2015 | 2 | 2 | 0 | 2 | 0 | 2 | 0 | 0 | 2 | 0 | 2 | 2 | 14/24 |
| Sutcliffe 2015 | 2 | 2 | 2 | 1 | 0 | 1 | 0 | 0 | 2 | 0 | 2 | 2 | 14/24 |
| Williamsson 2015 | 2 | 2 | 2 | 2 | 0 | 2 | 0 | 0 | 2 | 0 | 2 | 2 | 16/24 |
| Morales Soriano 2015 | 2 | 2 | 2 | 2 | 0 | 2 | 0 | 0 | 2 | 0 | 2 | 2 | 16/24 |
| Joliat 2015 | 2 | 2 | 2 | 2 | 0 | 1 | 0 | 0 | 2 | 0 | 2 | 2 | 15/24 |
| Bai 2016 | 2 | 2 | 2 | 2 | 0 | 1 | 0 | 0 | 2 | 0 | 2 | 2 | 15/24 |
| Zouros 2016 | 2 | 2 | 2 | 2 | 0 | 2 | 0 | 0 | 2 | 0 | 2 | 2 | 16/24 |
| Su 2017 | 2 | 2 | 2 | 2 | 0 | 1 | 0 | 0 | 2 | 0 | 2 | 2 | 13/24 |
| Perinela 2019 | 2 | 2 | 2 | 2 | 0 | 1 | 0 | 2 | 2 | 2 | 2 | 2 | 19/24 |
| ①A clearly stated aim；②Inclusion of consecutive patients；③Prospective collection of data；④Endpoints appropriate to the aim of the study；⑤Unbiased assessment of the study endpoint；⑥Follow-up period appropriate to the aim of the study；⑦Loss to follow up less than 5%；⑧Prospective calculation of the study size；⑨An adequate control group；⑩Contemporary groups；⑪Baseline equivalence of groups；⑫Adequate statistical analyses. The items are scored 0 (not reported), 1 (reported but inadequate) or 2 (reported and adequate). | | | | | | | | | | | | | |

| **Table S5 Results of Sensitivity Analysis** | | | | | |
| --- | --- | --- | --- | --- | --- |
| **Study** | **OR** | **95%CI** | **P Value** | **Heterogeneity**  **P Value** | **I^2^%** |
| Overall effect | 0.62 | 0.53-0.74 | <0.00001 | 0.23 | 18 |
| Exclude Kennedy 2007 | 0.62 | 0.52-0.73 | <0.00001 | 0.20 | 22 |
| Exclude Vanounou2007 | 0.62 | 0.52-0.74 | <0.00001 | 0.19 | 23 |
| Exclude Balzano 2008 | 0.62 | 0.52-0.75 | <0.00001 | 0.19 | 23 |
| Exclude Abu Hilal 2013 | 0.63 | 0.53-0.74 | <0.00001 | 0.23 | 19 |
| Exclude Kobayashi 2014 | 0.64 | 0.54-0.76 | <0.00001 | 0.26 | 16 |
| Exclude Braga 2014 | 0.61 | 0.51-0.73 | <0.00001 | 0.21 | 21 |
| **Exclude Coolsen 2014** | **0.60** | **0.51-0.69** | **<0.00001** | **0.46** | **0** |
| Exclude Shao 2015 | 0.65 | 0.54-0.77 | <0.00001 | 0.27 | 15 |
| Exclude Sutcliffe 2015 | 0.63 | 0.53-0.75 | <0.00001 | 0.19 | 23 |
| Exclude Williamsson 2015 | 0.62 | 0.52-0.73 | <0.00001 | 0.21 | 21 |
| Exclude Morales Soriano 2015 | 0.63 | 0.54-0.75 | <0.00001 | 0.26 | 16 |
| Exclude Joliat 2015 | 0.63 | 0.53-0.75 | <0.00001 | 0.21 | 21 |
| Exclude Bai 2016 | 0.62 | 0.52-0.73 | <0.00001 | 0.20 | 21 |
| Exclude Zouros 2016 | 0.63 | 0.53-0.75 | <0.00001 | 0.19 | 23 |
| Exclude Su 2017 | 0.63 | 0.54-0.74 | <0.00001 | 0.28 | 15 |
| Exclude Takagi 2019 | 0.63 | 0.54-0.74 | <0.00001 | 0.29 | 14 |
| Exclude Perinela 2019 | 0.61 | 0.52-0.72 | <0.00001 | 0.29 | 14 |
| Exclude Hwang 2019 | 0.61 | 0.51-0.72 | <0.00001 | 0.25 | 17 |
| OR=odds ratio, WMD=weighted mean difference, CI=confidence interval | | | | | |
